# Surgical Protocols before and after COVID-19—A Narrative Review

**DOI:** 10.3390/vaccines11020439

**Published:** 2023-02-14

**Authors:** Sahana Shivkumar, Vini Mehta, Sunil Kumar Vaddamanu, Urvashi A. Shetty, Fahad Hussain Alhamoudi, Maram Ali M. Alwadi, Lujain Ibrahim N. Aldosari, Abdulkhaliq Ali F. Alshadidi, Giuseppe Minervini

**Affiliations:** 1 Department of Public Health Dentistry, Peoples College of Dental Sciences & Research Centre, People University, Bhopal 462037, India; 2 Department of Public Health Dentistry, Dr. D.Y. Patil Dental College and Hospital, Dr. D.Y. Patil Vidyapeeth, Pimpri, Pune 411018, India; 3 Department of Dental Technology, College of Applied Medical Sciences, King Khalid University, Abha 62529, Saudi Arabia; 4 Department of Oral and Maxillofacial Pathology and Oral Microbiology, AB Shetty Memorial Institute of Dental Sciences, NITTE (Deemed to Be University), Mangalore 575018, India; 5 Dental Health Department, College of Applied Medical Sciences, King Saud University, Riyadh 11451, Saudi Arabia; 6 Prosthodontics Department, College of Dentistry, King Khalid University, Abha 61421, Saudi Arabia; 7 Multidisciplinary Department of Medical–Surgical and Dental Specialties, University of Campania Luigi Vanvitelli, 80138 Naples, Italy

**Keywords:** COVID-19, surgical guidelines, surgical practice, surgical protocol

## Abstract

The COVID-19 epidemic has affected not only people’s daily lives but also the working methods of clinicians, surgical procedures, open/minimally invasive procedures, operating room management, patient and healthcare worker safety, education and training. The main objective of this study was to review selected articles and determine the changes in the general surgery protocols/procedures before and after the emergence of the COVID-19 pandemic. The literature was carried out in PubMed-Medline, Cochrane Library, Embase, Scopus and Google Scholar. The terms utilised for the searches were “SARS-CoV-2”, “Surgery”, “COVID-19”, “Surgical protocol”, “Surgical recommendations” and “before and after”. A total of 236 studies were identified, out of which 41 studies were included for data extraction. Significant changes in all the articles were observed with respect to the surgeries done before, during and after the COVID-19 pandemic. Specifically, the number of elective surgeries were considerably fewer in comparison to the pre-pandemic period. Since the COVID-19 pandemic started, hospitals all throughout the world have conducted significantly fewer procedures, particularly elective/non-urgent surgeries.

## 1. Introduction

The coronavirus disease 2019 (COVID-19), which first appeared in late December 2019, spread quickly around the world causing millions of deaths since then and was designated a pandemic in March 2020 [1]. Due to the overabundance of contagious individuals and a newly discovered disease, the COVID-19 pandemic caused detrimental effects on all the facets of healthcare [2]. Hospitals and other medical facilities were overrun by sick people displaying a variety of symptoms and signs [3,4,5], making it impossible for them to perform a variety of treatments and surgeries while still maintaining the well-being of the patients and personnel. Therefore, a global call to postpone procedures appeared essential for as many people as possible [6,7].

The outbreak also had a significant impact on the surgeons and patients who needed surgical care. The patients and surgeons must have a special and close relationship to provide care for patients with surgical disease as telehealth cannot take the place of this interaction and contact [8]. As a result, the surgical workforce had experienced different difficulties during the COVID-19 pandemic than non-surgical specialists.

The best way to protect medical professionals and patients, the ability to effectively manage the delivery of healthcare provisions, the detrimental effects of diseases on patients, the economic toll that the pandemic will take on health care systems, the control of the inadequate staffing, the impact on education, investigations and research advancement and the psychological strain on all the people concerned are a few of the key topics discusses. A particular fact that has remained constant before and after the pandemic is that a healthy and effective surgical workforce is first and foremost required to provide surgical treatment. To accomplish this, all medical personnel must be well secured. Due to a shortage in adequate protective gear in the early phases of the pandemic, numerous medical systems encountered difficulties. The ability to protect the workforce improved along with supply chains and equipment accessibility, considering that the novel coronavirus has become endemic. It was established through thorough research investigations that everyone would benefit from taking widespread pandemic preparations. This entailed keeping a physical distance wherever possible, using a mask that fit snugly over the nose and mouth, often washing one’s hands, donning gloves while interacting with patients, routinely disinfecting surfaces and wearing eye protection throughout all patient interactions [9].

Organizations around the world and in the healthcare sector have been progressively returning to normal as the virus has been confined. One of the first actions of this return to normal was to resume surgery as many patients had experienced lengthy delays [10]. As a result, surgeries have slowly resumed in various hospitals, and there are now more invasive procedures performed globally. A lengthy waiting list in various operations would be anticipated by continuing to ease lockdown limitations and reduce the exponential growth of the viral spread and associated mortality [11,12]. Evidently, this pandemic has no end in sight, and the virus will continue to dominate the healthcare system. Given that certain precautions seemed to be taken for the safety and protection of the patients, there will be a transfer between the risk of surgical intervention during the pandemic and further delays or cancellations. This consequently has led to the adoption of different surgical practices/strategies in the wake of the changes employed by hospitals and healthcare facilities around the world due to the pandemic and the stresses brought on surgeons/clinicians/caregivers.

Hence, by the means of this review, we aimed to select relevant studies and determine the changes (if any) that have taken place with respect to performing surgeries and surgical protocols before and after the COVID-19 pandemic.

## 2. Materials and Methods

### 2.1. Focused Question

What are the changes in the surgical aspects of interventions prior to and after the occurrence of the COVID-19 pandemic?

The following types of literature were deemed eligible for inclusion: prospective studies, clinical trials and observational studies.

### 2.2. Literature Search

The entire search process was conducted independently by two investigators. An electronic search was conducted on the following research databases: PubMed-Medline, Cochrane Library Embase and Scopus. Furthermore, Google Scholar was used to search grey literature (newsletters, technology assessment reports, patients and speeches) focusing on surgical interventions before and after COVID-19. A literature search was carried out from the inception of COVID-19, i.e., from 31 December 2019 until 31 December 2022. The medical subject headings (MeSH) were: [SARS-CoV-2 OR Surgery OR COVID-19] AND [Coronavirus] AND [Surgical protocol OR Surgical recommendations]. The reference lists of the included articles were scanned to find additional studies meeting our inclusion criteria. Any disagreements were solved by discussion. An inter-examiner reliability score (Kappa score) was calculated to gauge the agreeability between the examiners. Any disagreements were solved by discussion. Google Translate was used to attempt the translation of the studies not in English.

### 2.3. Exclusion Criteria

- Case reports and case series were excluded.
- Articles which did not describe in detail about surgical interventions were excluded.

### 2.4. Selection of Studies

Two reviewers independently assessed the eligibility of the articles. In the event that the authors disagreed, the inclusion a third reviewer was sought for confirmation and consensus.

### 2.5. Data Extraction

Two investigators tabulated the data independently based on the surgical interventions before and after COVID-19 in different specialties.

### 2.6. Methodological Quality Appraisal

No formal assessment of the methodological quality of the assessment was done for the included studies.

## 3. Results and Discussion

### 3.1. Literature Search

**Figure 1:**
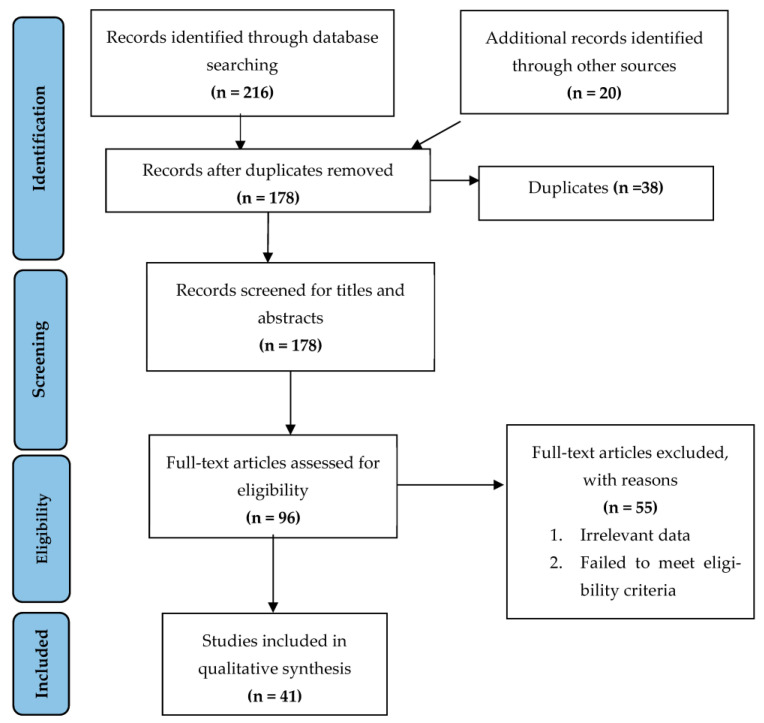
Flowchart summarizing the article selection process (*n—*number of studies).

After extensive searching, a total of 236 studies were identified, out of which 38 were duplicates. The remaining 178 studies underwent title and abstract screenings, and 96 studies were selected for a full text screening. Fifty-five studies were excluded after the full text screening. Thus, a total of 41 studies that met our inclusion criteria were processed for data extraction. The Kappa score was calculated as 0.83. The literature search process is illustrated as a PRISMA flow diagram (Figure 1).

For the sake of comprehensiveness and convenience, the results will be elaborated as per the various fields of surgery. Table 1 presents the list of the articles chosen based on the fields. We have included various surgical fields and discussed briefly about the changes in the surgical interventions due to COVID-19. Most of the articles (75.6%) described the surgical interventions after COVID-19 while 24.4% of the articles compared the surgical interventions before and after COVID-19 (Table 2).

### 3.2. Changes in Ocular/Ophthalmic Surgeries

The equipment and resources need to be allocated significantly for surgical procedures, especially the ones required for carrying out ocular procedures both in operatory and in post-surgical facilities. Thus, in early stages of the pandemic, it made sense to restrict operative capabilities to only emergency procedures. To compare the pandemic and pre-pandemic periods, Cetinkaya et al. [13] investigated the demographics of ophthalmic outpatients and cataract surgery hospitalizations in an ophthalmology clinic for tertiary care centre from April to June 2020. The number and distribution of the different surgical operations in each group performed amid the COVID-19 pandemic served as the primary objective. They found that, in 2020, 116 operations in total were completed. The same period of the year in 2019 saw 873 surgeries completed, which is an 86.7% drop during the pandemic. While Group A surgeries comprised 10.3% of all surgeries in 2019, they comprised 25.9% of all surgeries in 2020. Additionally, the frequency of Group B procedures rose from 5.4% to 17.24%. This huge decline in surgical procedures during the time of the pandemic is an observation by another study done by Shabto et al. [14]. From 17 February to 15 March to 16 March to 12 April 2020, they discovered a drop in total procedures from 87 to 34. In 7 days of the surgical orders being placed, the number of urgent cases decreased from 26 to four and the number of urgent cases within 21 days of the surgical orders decreased from 23 to 18. There were 62 surgeries between 16 March and 12 April 2019; 21 were emergent (34%) and 14 were urgent (23%). There were 68 surgeries between 16 March and 12 April 2018; 15 were emergent (22%) and 21 were urgent (30%). The American Academy of Ophthalmology (AAO) and the American Society of Retina Specialists (ASRS) established recommendations instructing ophthalmologists to halt surgeries considered elective or non-urgent, which may have contributed to the decline in the elective surgeries [15,16].

### 3.3. Changes in Urological Surgeries

Soytas et al. [17] published their investigation about comparing the effects of COVID-19 on urological practise. The changes among the outpatient examinations, non-surgical treatments and surgeries in the eight weeks prior to and following the pandemic were quantified by weeks. No significant differences between mean ages of the surgically treated patients before and after March 11 and their predisposing factors, which were 79 and 40, respectively. A total of 2309 and 868 patients underwent examinations, 173 and 94 underwent operations and 371 and 174 patients underwent non-surgical procedures, respectively. They showed that, even though the numbers had decreased, equivalent surgeries in standard urological practise had been performed with no infection or fatalities during the pandemic compared to the pre-pandemic period by taking precautions, even though the numbers had fallen.

### 3.4. Changes in Neurological Surgeries

A study examined the amount and type of neurosurgery patients that had been treated at two teaching hospitals in Egypt under the pandemic’s restrictive measures [18]. With the proportion of urgent procedures rising from 46 to 69% of all surgeries and the proportion of elective surgeries falling from 54 to 31% of all neurosurgeries, according to the study, the number of surgeries had decreased by 38% during the lockdown (second quarter of 2020) compared to total number of surgeries in the 1st quarter of same year. Comparing the 2nd quarter of 2020 to that of the year before, similar variations were seen in the quantity and variety of procedures, indicating that the scope and nature of neurosurgical practises have drastically changed due to the pandemic. There was a noticeable decline in the overall cases, but no discernible change in the multitude of urgent surgeries.

### 3.5. Changes in Oncological Surgeries

Grani et al. [19], by means of a review report, assessed the 12 months before and after March 2020 to examine how the interruption of routine activity affected the characteristics of differentiated thyroid tumours discovered at the end of the pandemic. In this study, the cohort was divided into two groups: the first group consisted of the cases identified before the COVID-19 lockdown (March 2019–February 2020), while the second group comprised of the cases identified both during and following the lockdown (March 2020–February 2021). They discovered that fewer procedures (around 34%) were conducted during the lockdown time, with no significant difference among both groups per the demographic characteristics such as age, gender, or clinical risk factors. However, there were fewer operations considered benign thyroid illness, which significantly decreased the incidence of accidentally discovered microcarcinomas. This was also shown in the distribution of the pre-surgical cytological diagnosis, where the reports of malignancy were more frequently found than those of benign or ambiguous conditions. The authors noted that after considering the risk classification and patient requirements as advised by a rapid consensus assertion released by the research organizations, thyroid procedures in Italy were postponed commencing in March 2020 [20]. A decrease in the cytological evaluations and diagnostic operations for benign or ambiguous lesions was, consequently, observed in the multicentre evaluations conducted throughout Italy [21,22].

### 3.6. Changes in Obstetrics/Gynaecological Surgeries

The research by Spurlin et al. [23] evaluated the impact of the COVID-19 pandemic in emergency wards (ER) of obstetrics-gynaecology (OC-GNY) departments in an institute in New York. According to the findings, gynaecology (GNY) surgeries and emergency room consultations declined throughout the pandemic, whereas obstetric (OC) surgeries remained steady. The proportions of the OC-GNY ER consultations, GNY surgeries and OC surgeries in comparison to all the ER consultations, surgeries and labour and delivery patients were 1.87 percent, 13.8 percent and 54.6% in the pre-COVID-19 time frame (1 February–15 March) vs. 1.53 percent, 21.3% and 79.7 percent during the pandemic (March 16–April 15), hence representing no significant difference in the proportions of the OC-GNY consults and GYN surgeries before and during COVID-19, with a significant increase in OC surgeries. With increasing proportions of emergency procedures for ectopic pregnancy, miscarriage and cancer concerns, the distribution of GNY surgical case categories underwent a dramatic change throughout pandemic. The gynaecological procedures performed and the cases treated were both, according to authors, impacted by institutional regulations [24] that prohibited elective surgery during the pandemic.

### 3.7. Changes in Orthopaedic Surgeries

Cengiz [25] documented his findings in a study where he examined the volume of the orthopaedic operations performed at a private institute before and after the COVID-19 pandemic. The difference in the number of orthopaedic procedures between the pre-COVID-19 era and the COVID-19 era served as the study’s primary outcome measure. The total number of surgeries (613 vs. 526), arthroplasty procedures (132 vs. 88), emergency surgeries in children (82 vs. 49) and tumour surgeries (44 vs. 27) all significantly decreased between the pre-COVID-19 and COVID-19 eras. The number of orthopaedic surgeries, including those connected to trauma and cancer, significantly decreased due to the COVID-19 pandemic measures implemented in Turkey. Their findings also reported that, during the pandemic time, there were significant reductions in patients admitted for tumour surgery, which could indicate that patients had less access to healthcare or that there were issues with the diagnosis and follow-up of the patients. Similar decreases in trauma patients have been documented in other nations. When the data were compared to the year 2018, Herngiou et al. [26] showed a comparable drop (up to 32%) in France due to the societal limitations brought on by the pandemic. Another Turkish study by Kalem et al. [27] found a 50% decrease in the patients suffering from traumatic injuries compared to the similar time frame before the pandemic.

### 3.8. Changes in Plastic Surgery Practices

Mortada et al. [28] reported a retrospective comparative study that evaluated how the COVID-19 pandemic affected the trends and traits of plastic surgeries in Riyadh, Saudi Arabia, at King Saud University. The COVID-19 pandemic, which ran from March–December 2020 with an equivalent pre-pandemic period, which was same time period in the year before, were both examined in their research. The pre-pandemic patient admissions totalled 479, while the pandemic admissions totalled 254, representing a 46.97% decrease in the admission frequency. In comparison to 2019, the median hospital stay was much shorter in 2020. This meant that there was a markedly higher frequency of urgent procedures and lower rates of elective procedures. The authors concluded that since the pandemic had started, fewer plastic procedures were performed. The pandemic’s long-term effect on the practises of plastic surgery, however, is still unknown, which necessitates more research in this regard. The significant reduction in craniofacial surgeries observed in this study also agrees with an investigation done by Kalantar et al. [29]. The investigators attributed this decrease in the riskiness of the surgical site because the pharynx and upper digestive tract are where sick people often have the highest virus loads [30]. On the other hand, a comparative study done in the US employing a similar methodological approach found fewer emergency cases were hospitalised after a cosmetic surgery than in 2019 [31].

### 3.9. Changes in Pre- and Post-Operative Complications

Yeganeh et al. [32], in their systematic review and meta-analysis, assessed the changes in the pre- and post-operative complications from the pre-pandemic and post-pandemic periods. After choosing 34 articles from an initial pool of 909 studies published between 1 January 2019 and 3 November 2021 using the Clavien–Dindo classification method for rating the surgical complications, 19,137 patients (3522 patients before and 15,615 patients after the COVID-19 pandemic) were evaluated in this review. They discovered that, even while surgical complications ranged from grade 1–4, post-operative death (grade 5) increased during the time of the pandemic compared to the levels prior to the outbreak. The decreased survival rate following surgery may be explained by a more advanced stage of cancer, a delay in therapy or the selection of the patients. This observation was shared in three more studies [33,34,35]. The influence of COVID-19 on surgical practise isn’t just confined to COVID-19 patients since other publications [33,34,35,36] attribute increased post-operative morbidity and mortality among COVID-19 patients. The meta-analysis of the data indicates that COVID-19-negative individuals who received surgery during the COVID-19 timeframe were similarly at a higher mortality risk. The studies in the literature database that is currently accessible corroborated a decline in the admission rates [37,38,39,40], which may be due to the patients’ hesitation to be admitted due to the fear of COVID-19. The indicators of the prognosis, such as the complications at the presentation, the severity of the trauma, and the pathologic findings, were strongly skewed in the COVID-19 era in favour of disease complexity and severity. The outcome bias can also be brought on by the changes in the admissions rate. On the one hand, some circumstances saw a considerable decline in elective procedures, which had a better prognosis. Conversely, fewer surgical admissions and tight COVID-19 protocols led to fewer operations and a delayed patient turnover, which may have contributed to the positive results. One of two studies indicated that serious problems in the COVID-19 pandemic era compared to the control cohort (representing the post-pandemic time) was one of the three studies out of the ten in the meta-analysis conducted by Yeganeh et al. [32] that suggested a significantly greater rate of post-operative complications [38] than the control cohort [39]. Both studies were carried out in Italy, (the 1st nation to encounter the SARS-COV-2 outbreak) during the 1st COVID-19 wave. Above all, these trials reported no claims for any COVID-19 screenings or prevention strategies. Panda et al. [41] discovered that the application of the protection strategies led to a reduction in the post-operative problems both at times before and after the pandemic, as well as a decreased rate in the complications in a matched pair analysis.

### 3.10. Changes in General Surgical Practices

A two-part series of studies documenting the changes in the surgical prioritisation was reported at the start and during the pandemic by Al-Jabir et al. [42,43]. Part 1 of this analysis [42] dealt with the surgical guidelines and broadly focused on the changes that hospitals and medical institutions around the world needed to accommodate/deal with the rise in the multitude of COVID-19 sufferers and their initially high rate of hospitalisations. They also noted that the COVID-19 pandemic at its inception had required an immediate repurposing and reorganisation of the global surgical workforce. Authorities, such as NHS England, were told to halt every non-urgent elective procedure for at least 3 months with the addition of advising hospitals to quickly discharge all medically fit patients [44]. Tao et al. suggested that laparoscopy would have to be avoided because artificial pneumoperitoneum could cause an increase in airway pressure, CO_2_ retention and a decrease in lung compliance that was not detrimental with the functions after the operative procedures. Laparoscopy is another surgical step that forms aerosols. Tao et al. [45] noted the virus detection in smoke generated from the surgical procedures. For reducing the hazards from pneumoperitoneum, Zheng et al. [46] described the knowledge of minimal invasion for surgery in China and Italy. They suggested using the lowest possible insufflation pressures and intraperitoneal suction. Additionally, the Royal Surgical Colleges of Great Britain had recommended that laparoscopy can be reserved for specific situations where it was clinically necessary and feasible, given the threat of the virus transmission to the healthcare personnel [54]. These guidelines were significantly different from the ones that the hospitals had followed in the pre-pandemic era, but such was the state of the healthcare centres around the globe that elective surgeries had to be postponed as hospitals battled an ever-increasing patient count when the COVID-19 outbreak began.

Part 2 of the study [43] discussed further about the surgical prioritisation as it was published when the pandemic was in full force during the mid-autumn of 2020. The cases of the incision and drainage of perianal/perirectal abscesses, necrotising pancreatitis, closed loop bowel obstructions, incarcerated hernias, bowel perforations, intestinal ischaemia, appendectomies, cholecystectomies, diverticulitis and emergency laparotomies where bleeding did not respond to the endoscopic/interventional radiology procedures were deemed non-elective. The surgeries involving bowel obstruction due to adhesions, uncomplicated appendectomy, cholecystectomy and cholelithiasis, pseudo-obstruction and adrenal cancer surgery were deferred according to the guidelines given by NHS England [55] and the American College of Surgeons [56].

By analysing the following elective procedures carried out between September 2018 and September 2021, Demir et al. [47] sought to determine impact of COVID-19 on a number of surgeries carried out before and after the pandemic for the upper gastrointestinal system, abdominal wall hernias, gallbladder surgeries and kidney transplantations. The date of 11 March 2020 was selected as the cut-off date for the two groups for dividing the samples into before and after COVID-19, and no elective procedures were postponed. Out of the 1420 samples that were treated for elective surgeries from September 2018–2021, before the COVID-19 group, there were 72% samples, and after the COVID-19 group there were 27.2% samples. Significant differences between these two groups were discovered. The surgical patients comprised 55.1% men and 44.9% women. The procedural steps published by the Ministry of Health of the Republic of Turkey established the operation groups A, B and C. Group A operations included major surgeries that required high levels of experience such as gastrectomy and esophagectomy, Group B operations were less difficult surgeries such as cholecystectomy and laparoscopic hernia repair and Group C were minor surgeries such as umbilical hernia repair. The authors observed that Group A operations comprised 372 (26.2%) of the total operations, Group B operations comprised 757 (53.3%) and Group C operations comprised 20.5%. Approx. 27.8% of the surgeries before the COVID-19 group were in Group A, 51.4% were in Group B and 20.8% were in Group C. Approx. 21.9% of the surgeries in the post-pandemic group fell into Group A, 58.4% fell into Group B and 19.7% fell into Group C. In total, 820 surgeries (57.8%) used the laparoscopic approach, while 600 (42.2%) used the open technique. In the pre-pandemic group, 581 (56.2%) and 452 (43.8%) of the surgeries used the laparoscopic approach, respectively. In the post-pandemic group, 239 (61.8%) laparoscopic procedures and 148 (38.2%) open procedures were carried out, indicating a substantial difference regarding the surgical groups of procedures carried out before and after the COVID-19 pandemic groups as well as the type of surgery performed. These observations agreed with another research conducted in India by Nasta et al. [48]. It can be argued, however, that the various surgeries, such as colorectal, hepatobiliary and breast surgery, might have given detailed information and unbiased findings and that carrying out a single-centre study could have introduced some bias as well.

### 3.11. Changes in Elective Surgeries

A study by Aashna et al. [49], focused on the changes in the operational guidelines over the duration of the pandemic and how these alterations continue to affect the services to patients and the patient experience as COVID-19 eased and progressively converted into an endemic entity. The healthcare system needed to prepare for a sizable increase in surgeries as the COVID-19 pandemic wound down because the majority of elective procedures were shifted to a later time. The COVIDSurg Collaborative study found that if countries had raised the usual volume of surgeries by 20% after the pandemic, it would have taken a median of 45 weeks to clear the delay in the procedures from 12 weeks of the peak pandemic [50]. According to various post-pandemic procedures, the surgical backlog at two of the busiest cardiac surgery programmes in Maryland would have been cleared in 1–8 months with an operational volume of 216–263% in a month [51]. It would have taken the US healthcare system 16 months to finish performing the backlog of postponed total knee arthroplasty surgery [52]. In contrast to the pre-pandemic era, they pointed out that elective surgeries do not need to be postponed in the post-pandemic era because they are considered “important surgeries” by the American College of Surgeons. By adhering to the standards and regulations set in place during the pandemic, many non-emergent surgeries, such as those for cancer treatment, might have been avoided or postponed without suffering serious effects. One study estimated that a delay of more than 4 months would result in an increase of over 10,000 colon cancer deaths [53]. The authors’ advice was to start by recommending the resumption of elective procedures considering their findings. Given that the maximum surgeries would have had a lengthy wait period, such organisations ought to have prioritised their waitlist on basis of the risk criteria such as the urgency of the treatment, the health of patient and the length of the delays from the consultation. It was already established that they demonstrated the safety of surgeries even in the presence of COVID-19, with no evidence of a heightened risk of infection following a surgery. However, they recommended designating a small number of surgical groups (comprising nursing staff, technicians, administrating staff and so on) for performing a nasopharyngeal swab on patient prior to surgery, verifying negative results before arriving, which will further ensure the highest ordinance in the cross-contamination and infection control both before and after the surgery. They also suggested giving patients pamphlets or information sheets outlining the practises after the surgeries, which will prevent the SARS-COV-2 spread (e.g., isolating, usage of face masks, hygiene measures and so on), something that wasn’t available in the run-up to the pandemic because of how little clinicians knew about the novel coronavirus at the time.

## 4. Limitations

It is necessary to take into account the content in this review as well as some significant constraints. The information offered in our review mainly focused on two or three phases, namely before the pandemic started and during and after the pandemic. Additionally, our interpretation of the findings may have been muddled by the heterogeneity of the study techniques, statistical approaches, sample sizes, demographic characteristics, geographic locations and publishing quality. Last but not least, many of the apparent relationships mentioned in this review have yet to be verified by further researchers or supported by reliable statistical techniques. However, it is critical to address the knowledge gaps in the field and identify the variables that may be indicative of the COVID-19 problems that surgeons/clinicians face while conducting surgeries (both elective and non-elective) and the call for further research in the context of the novel virus that is endemic in today’s world.

## 5. Conclusions

The number of surgeries, specifically elective/non-urgent surgeries, that hospitals around the world have performed has significantly reduced since the COVID-19 pandemic started. Most surgical protocols implemented in healthcare institutions have virtually remained the same since the virus has become endemic nearly everywhere. However, while this pandemic enforced hospitals to discontinue several beneficial treatments, it has also given clinicians a chance to concentrate on benefit–risk ratio of the surgical options.

## Figures and Tables

**Table 1:** List of the articles reviewed.

Surgical Field	Articles Included
Ocular/Ophthalmic	Cetinkaya et al. [13], Shabto J.M. [14], AA of Opthalmology [15], American Society of Retina Specialists [16]
Urology	Soytas M. et al. [17]
Neurology	Nabil M. et al. [18]
Oncology	Grani G. et al. [19], Puig-Domingo M. et al. [20], Medas F. et al. [21], Vigiliar E. [22]
OBG	Spurlin E.E. et al. [23], American College of Obstetricians and Gynecolgists [24]
Orthopaedic	Cengiz and Bertam [25], Hernigou J. et al. [26], Kalem et al. [27]
Plastic surgery	Mortada H. et al. [28], Kalantar et al. [29], Hormozi A. et al. [30], Andrews et al. [31]
Surgical complications	Yeganeh Farsi [32], Surgery Colloborative [33], Abbott T.E.F. [34], Knisely A. et al [35], Kaufmen E.J. et al. [36], Kumaira Fonseca [37], Tartaglia N. [38], Sartori A. [39], Yusirikala A. et al. [40], Panda [41]
General surgery	Al-Jabir A. [42], Al-Jabir. [43], Jacobucci G. [44], Tao K.X. et al. [45], Zheng MH et al. [46], Demir HB et al. [47], Nasta et al. [48]
Elective surgery	Mehta A. et al. [49], Farr S. et al. [50], Salenger R. et al. [51], Cisternas A.F. et al. [52], Larson D.W. et al. [53]

OBG: Obstetrics and gynaecology.

**Table 2:** Intervals of the studies conducted.

Intervals	Studies
After [32/41] (75.6%)	AA of Opthalmology [15], American Society of Retina Specialists [16], Grani G. et al. [19], Puig-Domingo M. et al. [20], Medas F. et al. [21], Vigiliar E. [22], Spurlin E.E. et al. [23], American College of Obstetricians and Gynecolgists [24], Cengiz and Bertam [25], Hernigou J. et al. [26], Kalem et al. [27], Hormozi A. et al. [30], Surgery Colloborative [33], Abbott T.E.F. [34], Kaufmen E.J. et al. [36], Kumaira Fonseca [37], Tartaglia N. [38], Sartori A. [39], Yusirikala A. et al. [40], Panda [41], Al-Jabir A. [42], Al-Jabir [43], Jacobucci G. [44], Tao K.X. et al. [45], Zheng M.H. et al. [46], Demir H.B. et al. [47], Nasta et al. [48], Mehta A. et al. [49], Farr S. et al. [50], Salenger R. et al. [51], Cisternas A.F. et al. [52], Larson D.W. et al. [53]
Before and after [10/41] (24.4%)	Cetinkaya et al. [13], Shabto J.M. [14], Soytas M. et al. [17], Nabil M. et al. [18], Mortada H. et al. [28], Kalantar et al. [29], Hormozi A. et al. [30], Andrews et al. [31], Yeganeh Farsi [32], Knisely A. et al. [35],

## Data Availability

Not applicable.

## References

[1] Zhu N., Zhang D., Wang W., Li X., Yang B., Song J., Zhao X., Huang B., Shi W., Lu R. (2020). A Novel Coronavirus from Patients with Pneumonia in China, 2019. N. Engl. J. Med..

[2] Sohrabi C., Alsafi Z., O’Neill N., Khan M., Kerwan A., Al-Jabir A., Iosifidis C., Agha R. (2020). World Health Organization declares global emergency: A review of the 2019 novel coronavirus (COVID-19). Int. J. Surg..

[3] World Health Organisation (2020). WHO Director-General’s Opening Remarks at the Media Briefing on COVID-19. https://www.who.int/dg/speeches/detail/whodirector-general-s-opening-remarks-at-the-media-briefing-on-covid-19-11-march-2020.

[4] Nicola M., O’Neill N., Sohrabi C., Khan M., Agha M., Agha R. (2020). Evidence based management guideline for the COVID-19 pandemic—Review article. Int. J. Surg..

[5] Nicola M., Alsafi Z., Sohrabi C., Kerwan A., Al-Jabir A., Iosifidis C., Agha M., Agha R. (2020). The socio-economic implications of the coronavirus pandemic (COVID-19): A review. Int. J. Surg..

[6] Kurihara H., Bisagni P., Faccincani R., Zago M. (2020). COVID-19 outbreak in Northern Italy: Viewpoint of the Milan area surgical community. J. Trauma: Inj. Infect. Crit. Care.

[7] Guan W.J., Ni Z.Y., Hu Y., Liang W.H., Qu C.Q., He J.X., Liu L., Shan H., Lei C.L., Hui D.S.C. (2020). Clinical Characteristics of coronavirus disease 2019 in China. N. Engl. J. Med..

[8] Monaghesh E., Hajizadeh A. (2020). The role of telehealth during COVID-19 outbreak: A systematic review based on current evidence. BMC Public Health.

[9] European Centre for Disease Prevention and Control (ECDC) (2020). Infection Prevention and Control and Preparedness for COVID-19 in Healthcare Settings. https://www.ecdc.europa.eu/en/publications-data/infection-prevention-and-control-andpreparedness-covid-19-healthcare-settings.

[10] Brindle M.E., Gawande A. (2020). Managing COVID-19 in Surgical Systems. Ann. Surg..

[11] COVIDSurg Collaborative (2020). Global guidance for surgical care during the COVID-19 pandemic: Surgical care during the COVID-19 pandemic. Br. J. Surg..

[12] (2014). Monitor, International Comparisons of Selected Service Lines in Seven Health Systems. Annex 3—Review of Service Lines: Critical Care. https://assets.publishing.service.gov.uk/government/uploads/system/uploads/attachment_data/file/382845/Annex_3_Critical_Care1.pdf.

[13] Cetinkaya Y.F. (2022). Ophthalmic surgeries before and during the covid-19 outbreak in a tertiary hospital. Int. Ophthalmol..

[14] Shabto J.M., Faaborg-Andersen C., O’Keefe G.A. (2022). The impact of COVID-19: Variations in volumes and characteristics of retina surgeries. BMC Surg..

[15] American Academy of Ophthalmology New Recommendations for Urgent and Nonurgent Patient Care. https://www.aao.org/headline/new-recom%20mendations-urgent-nonurgent-patient-care.

[16] American Society of Retina Specialists ASRS Releases Guidelines to Help Retina Practices Navigate COVID-19 Pandemic. https://www.asrs.org/clinical/clinical-updates/1962/asrs-releases-guidelines-to-help-retina-practices-navigate-covid-19-pandemic.

[17] Soytas M., Boz M.Y., Guzelburc V., Calik G., Horuz R., Akbulut Z., Albayrak S. (2020). Comparison of before and after COVID-19 urology practices of a pandemic hospital. Turk. J. Urol..

[18] Nabil M., Dorrah M., Sharfeldin A., Abaza H. (2022). Impact of COVID-19 pandemic on the neurosurgical practice in Egypt. Egypt. J. Neurosurg..

[19] Grani G., Ciotti L., Del Gatto V., Montesano T., Biffoni M., Giacomelli L., Sponziello M., Pecce V., Lucia P., Verrienti A. (2022). The COVID-19 outbreak and de-escalation of thyroid cancer diagnosis and treatment. Endocrine.

[20] Puig-Domingo M., Marazuela M., Giustina A. (2020). COVID-19 and endocrine diseases. A statement from the European Society of Endocrinology. Endocrine.

[21] Medas F., Ansaldo G.L., Avenia N., Basili G., Boniardi M., Bononi M., Bove A., Carcoforo P., Casaril A., Cavallaro G. (2021). The THYCOVIT (Thyroid Surgery during COVID-19 pandemic in Italy) study: Results from a nationwide, multicentric, case-controlled study. Updat. Surg..

[22] Vigliar E., Pisapia P., Iacovo F.D., Alcaraz-Mateos E., Alì G., Ali S.Z., Baloch Z.W., Bellevicine C., Bongiovanni M., Botsun P. (2022). COVID-19 pandemic impact on cytopathology practice in the post-lockdown period: An international, multicenter study. Cancer Cytopathol..

[23] Spurlin E.E., Han E.S., Silver E.R., May B.L., Tatonetti N.P., Ingram M.A., Jin Z., Hur C., Advincula A.P., Hur H.-C. (2020). Where Have All the Emergencies Gone? The Impact of the COVID-19 Pandemic on Obstetric and Gynecologic Procedures and Consults at a New York City Hospital. J. Minim. Invasive Gynecol..

[24] American College of Obstetricians and Gynecologists Joint Statement on Elective Surgeries. https://www.acog.org/news/newsreleases/2020/03/joint-statement-on-elective-surgeries.

[25] Cengiz B. (2022). The impact of COVID-19-related social restriction and containment measures on admissions for orthopedic surgery in a private hospital: Cross-sectional data from a secondary healthcare provider in Turkey. Med. Sci. | Int. Med. J..

[26] Hernigou J., Morel X., Callewier A., Bath O., Hernigou P. (2020). Staying home during “COVID-19” decreased fractures, but trauma did not quarantine in one hundred and twelve adults and twenty-eight children and the “tsunami of recommendations” could not lockdown twelve elective operations. Int. Orthop..

[27] Kalem M., Kocaoglu H., Merter A., Karaca M.O., Özbek E.A., Kinik H.H. (2021). Effects of COVID-19 pandemic curfew on orthopedic trauma in a tertiary care hospital in Turkey. Acta Orthop. Traumatol. Turc..

[28] Mortada H., Alawaji Z.H., Aldihan R.A., Alkuwaiz L.A., Alshaalan S.F., Kattan A.E. (2022). Impact of the Coronavirus Disease 2019 Pandemic on the Patterns and Characteristics of Plastic Surgery Practice: A Retrospective Comparative Study of Before and During the Pandemic. Cureus.

[29] Kalantar-Hormozi A., Habibzadeh Z., Yavari M., Mousavizadeh S.M., Hassanpour S.E., Motamed S., Rouientan A., Mozafari N., Shahrokh S., Mohammadsadeghi S. (2021). Impact of COVID-19 pandemic on plastic surgery activities and residency programs in a tertiary referral centre in Iran. Eur. J. Plast. Surg..

[30] Andrews B.T., Garg R., Przylecki W., Habal M. (2020). COVID-19 Pandemic and its Impact on Craniofacial Surgery. J. Craniofacial Surg..

[31] Paiva M.M., Rao V.M., Spake C.S.M., King V.A., Crozier J.W.M., Liu P.Y., Woo A.S., Schmidt S.T.M., Kalliainen L.K.M. (2020). The Impact of the COVID-19 Pandemic on Plastic Surgery Consultations in the Emergency Department. Plast. Reconstr. Surg.—Glob. Open.

[32] Farsi Y., Shojaeian F., Ahmad Safavi-Naini S.A., Honarvar M., Mohammadzadeh B., Nasiri M.J. (2022). The comparison of Post-Operative Complications pre Covid era versus during Covid-Era based on Clavien-Dindo-classification: A Systematic Review and Meta-Analysis. medRxiv.

[33] Collaborative COVID (2020). COVID Surg Collaborative. Mortality and pulmonary complications in patients undergoing surgery with perioperative SARS-CoV-2 infection: An international cohort study. Lancet.

[34] Abbott T.E., Fowler A.J., Dobbs T.D., Gibson J., Shahid T., Dias P., Akbari A., Whitaker I.S., Pearse R.M. (2021). Mortality after surgery with SARS-CoV-2 infection in England: A population-wide epidemiological study. Br. J. Anaesth..

[35] Knisely A., Ni Zhou Z., Wu J., Huang Y., Holcomb K., Melamed A., Advincula A.P., Lalwani A., Khoury-Collado F., Tergas A.I. (2020). Perioperative Morbidity and Mortality of Patients With COVID-19 Who Undergo Urgent and Emergent Surgical Procedures. Ann. Surg..

[36] Kaufman E.J.M., Ong A.W., Cipolle M.D.M., Whitehorn G.B., Ratnasekera A.D., Stawicki S.P.M., Martin N.D. (2021). The impact of COVID-19 infection on outcomes after injury in a state trauma system. J. Trauma Inj. Infect. Crit. Care.

[37] Kumaira Fonseca M., Trindade E.N., Costa Filho O.P., Nácul M.P., Seabra A.P. (2020). Impact of COVID -19 Outbreak on the Emergency Presentation of Acute Appendicitis. Am. Surg..

[38] Tartaglia N., Pavone G., Lizzi V., Vovola F., Tricarico F., Pacilli M., Ambrosi A. (2020). How emergency surgery has changed during the COVID-19 pandemic: A cohort study. Ann. Med. Surg..

[39] Sartori A., Podda M., Botteri E., Passera R., Agresta F., Arezzo A., CRAC Study Collaboration Group (2021). Appendectomy during the COVID-19 pandemic in Italy: A multicenter ambispective cohort study by the Italian Society of Endoscopic Surgery and new technologies (the CRAC study). Updates Surg..

[40] Vusirikala A., Saleh M., Laurent E., del Castillo T., Kuzhupilly R.R., Fahmy A., Tsekes D. (2021). Restarting Elective Orthopaedic Surgery During the COVID-19 Pandemic: Lessons Learned. Cureus.

[41] Panda S., Vig S., Singh C.A., Konkimalla A., Thakar A., Sakthivel P., Sikka K., Kumar R., Bhatnagar S., Mohan A. (2021). Head and Neck Surgery During COVID-19 Pandemic: Experience from a Tertiary Care in India. Indian J. Surg. Oncol..

[42] Al-Jabir A., Kerwan A., Nicola M., Alsafi Z., Khan M., Sohrabi C., O’Neill N., Iosifidis C., Griffin M., Mathew G. (2020). Impact of the Coronavirus (COVID-19) pandemic on surgical practice—Part 1. Int. J. Surg..

[43] Al-Jabir A., Kerwan A., Nicola M., Alsafi Z., Khan M., Sohrabi C., O’Neill N., Iosifidis C., Griffin M., Mathew G. (2020). Impact of the Coronavirus (COVID-19) pandemic on surgical practice—Part 2 (surgical prioritisation). Int. J. Surg..

[44] Iacobucci G. (2020). COVID-19: All non-urgent elective surgery is suspended for at least three months in England. BMJ.

[45] Tao K.X., Zhang B.X., Zhang P., Zhu P., Wang G.B., Chen X.P., General Surgery Branch of Hubei Medical Association, General Surgery Branch of Wuhan Medical Association (2020). Recommendations for general surgery clinical practice in 2019 coronavirus disease situation. Zhonghua Wai Ke Za Zhi.

[46] Zheng M.H., Boni L., Fingerhut A. (2020). Minimally Invasive Surgery and the Novel Coronavirus Outbreak: Lessons Learned in China and Italy. Ann. Surg..

[47] Demir H.B., Korucuk E., Miftari A., Turk Y. (2022). Have General Surgery Practices Decreased During the COVID-19 Pandemic?. Cureus.

[48] Nasta A.M., Goel R., Kanagavel M., Easwaramoorthy S. (2020). Impact of COVID-19 on General Surgical Practice in India. Indian J. Surg..

[49] Mehta A., Awuah W.A., Ng J.C., Kundu M., Yarlagadda R., Sen M., Nansubuga E.P., Abdul-Rahman T., Hasan M.M. (2022). Elective surgeries during and after the COVID-19 pandemic: Case burden and physician shortage concerns. Ann. Med. Surg..

[50] Farr S., Berry J.A., Berry D.K., Marotta D.A., Buckley S.E., Javaid R., Jacqueline D.M., Magargee C.E., Ferrouge L.M., Rogalska A.M. (2021). The Impact of the COVID-19 Pandemic on Resident Physicians Well-Being in the Surgical and Primary Care Specialties in the United States and Canada. Cureus.

[51] Salenger R., Etchill E., Ad N., Matthew T., Alejo D., Whitman G., Lawton J., Lau C.L., Gammie C., Gammie J. (2020). The surge after the surge: Cardiac surgery post-COVID-19. Ann. Thorac. Surg..

[52] Cisternas A.F., Ramachandran R., Yaksh T.L., Nahama A. (2020). Unintended consequences of COVID-19 safety measures on patients with chronic knee pain forced to defer joint replacement surgery. PAIN Rep..

[53] Larson D.W., El Aziz M.A.A., Mandrekar J.N. (2020). How Many Lives Will Delay of Colon Cancer Surgery Cost During the COVID-19 Pandemic? An Analysis Based on the US National Cancer Database. Mayo Clin. Proc..

[54] Association of Surgeons of Great Britain & Ireland, Association of coloproctology of Great Britain & Ireland, Association of Upper Gastrointestinal Surgeons, Royal College of Surgeons of Edinburgh, Royal College of Surgeons of England, Royal College of Physicians and Surgeons of Glasgow, Royal College of Surgeons in Ireland (2020). Updated General Surgery Guidance on COVID-19, 2nd Revision. https://www.rcsed.ac.uk/news-public-affairs/news/2020/april/updated-general-surgeryguidance-on-covid-19-2nd-revision-7th-april-2020.

[55] (2020). NHS England, Royal College of Surgeons of England, Royal College of Surgeons in Ireland, Royal college of surgeons of Edinburgh, Royal College of Physicians and Surgeons of Glasgow, Clinical Guide to Surgical Prioritisation during the Coronavirus Pandemic. https://www.england.nhs.uk/coronavirus/wp-content/uploads/sites/52/2020/03/C0221-specialty-guide-surgical-prioritisation-v1.pdf.

[56] American College of Surgeons (2020). COVID-19 Guidelines for Triage of Emergency General Surgery Patients. https://www.facs.org/covid-19/clinicalguidance/elective-case/emergency-surgery.

